# Meta-Analysis Using a Novel Database, miRStress, Reveals miRNAs That Are Frequently Associated with the Radiation and Hypoxia Stress-Responses

**DOI:** 10.1371/journal.pone.0080844

**Published:** 2013-11-14

**Authors:** Laura Ann Jacobs, Findlay Bewicke-Copley, Mark Graham Poolman, Ryan Charles Pink, Laura Ann Mulcahy, Isabel Baker, Ellie-May Beaman, Travis Brooks, Daniel Paul Caley, William Cowling, James Michael Stevenson Currie, Jessica Horsburgh, Lottie Kenehan, Emma Keyes, Daniel Leite, Davide Massa, Adam McDermott-Rouse, Priya Samuel, Hannah Wood, Munira Kadhim, David Raul Francisco Carter

**Affiliations:** 1 Department of Biological and Medical Sciences, Oxford Brookes University, Oxford, United Kingdom; 2 Canada’s Michael Smith Genome Sciences Centre, BC Cancer Agency, Vancouver, British Columbia, Canada; East Carolina University, United States of America

## Abstract

Organisms are often exposed to environmental pressures that affect homeostasis, so it is important to understand the biological basis of stress-response. Various biological mechanisms have evolved to help cells cope with potentially cytotoxic changes in their environment. miRNAs are small non-coding RNAs which are able to regulate mRNA stability. It has been suggested that miRNAs may tip the balance between continued cytorepair and induction of apoptosis in response to stress. There is a wealth of data in the literature showing the effect of environmental stress on miRNAs, but it is scattered in a large number of disparate publications. Meta-analyses of this data would produce added insight into the molecular mechanisms of stress-response. To facilitate this we created and manually curated the miRStress database, which describes the changes in miRNA levels following an array of stress types in eukaryotic cells. Here we describe this database and validate the miRStress tool for analysing miRNAs that are regulated by stress. To validate the database we performed a cross-species analysis to identify miRNAs that respond to radiation. The analysis tool confirms miR-21 and miR-34a as frequently deregulated in response to radiation, but also identifies novel candidates as potentially important players in this stress response, including miR-15b, miR-19b, and miR-106a. Similarly, we used the miRStress tool to analyse hypoxia-responsive miRNAs. The most frequently deregulated miRNAs were miR-210 and miR-21, as expected. Several other miRNAs were also found to be associated with hypoxia, including miR-181b, miR-26a/b, miR-106a, miR-213 and miR-192. Therefore the miRStress tool has identified miRNAs with hitherto unknown or under-appreciated roles in the response to specific stress types. The miRStress tool, which can be used to uncover new insight into the biological roles of miRNAs, and also has the potential to unearth potential biomarkers for therapeutic response, is freely available at http://mudshark.brookes.ac.uk/MirStress.

## Introduction

When faced with an environmental stressor an organism can either extricate itself from the situation or adapt by other means. When individual cells encounter such stresses they are often unable to escape, and so a number of biological mechanisms have evolved to help cells cope with potentially cytotoxic changes in their environment. Stressful stimuli or ‘stresses’ may include extremes of temperature, chemical exposure, hypoxia, radiation or nutrient stress [1]. Cells within a multi-cellular organism employ mechanisms to adapt to the change, repair the damage caused by the stressor, or undergo apoptosis to protect the organism [2]. Organisms are often exposed to environmental pressures, such as radiation exposure, which affect homeostasis, and so it is important to understand the biological basis of stress-response.

Key survival mechanisms of cells include the heat shock response [3] or the unfolded protein response (UPR) [4]. In reaction to most stresses there is a swift intervention to normal protein production within the cell. Though global translation is often reduced following stress-induction [5], translation of specific transcripts is up-regulated [6]. Cellular material that is deemed unnecessary, including various transcripts, are degraded [7]. Stress granules (SG) form inside cells, which appear to sequester specific transcripts along with ribosomal proteins; SGs are dispersed soon after the stimulus is removed [8]. Steady-state levels of different mRNAs can be affected by post-transcriptional mechanisms [9]. Post-transcriptional regulation, for example by interaction between mRNAs and binding proteins [7], affords a potentially more rapid response to stress [1]. 

Recent findings from genome analysis consortia have indicated that most organisms produce a myriad of non-coding RNAs. Whilst the role of the majority of this transcriptional output remains controversial, there are an increasing number of long [10,11] and short [12] non-coding RNAs with a demonstrated functional role in health or disease. miRNAs are short (approximately 22 nt) RNAs that can interact with the 3’UTRs of target mRNAs, resulting in translational repression and mRNA degradation [13]. Because the interaction between a miRNA and its target is based on a small region of ~7 nucleotides, which does not need to match perfectly, a single miRNA can affect the expression of many genes simultaneously [14]. Given the importance of regulating mRNA stability in response to stress it is unsurprising that miRNAs also show a dynamic response when cells encounter a perturbagen [15]. Indeed, it has been suggested that miRNAs may ultimately tip the balance between continued cytorepair and induction of apoptosis [1,16]. 

The importance of miRNAs in a cellular and organismal context remains controversial. Although miRNAs were first discovered through their phenotypic effect on *C. elegans* [17], deletion of various miRNAs has no apparent consequence [18,19]. This contradicts the functional importance of these miRNAs implied by their often high sequence conservation. This paradox has, at least in part, been resolved by studies looking at the effects of miRNAs in response to stress. Indeed, in some cases the phenotypic effects of the miRNA deletion only became apparent after the organism is exposed to environmental stress. For example, miR-214 was shown to be a marker of cardiac stress [20], yet knocking out miR-214 in mice had no effect on physiology under normal conditions [21]. However, when these mice were stressed by ischemia/reperfusion injury they exhibited increased apoptosis of cardiac cells and decreased overall survival [21]. Similarly, miR-7 mutant flies have a wild-type phenotype under normal conditions, but when exposed to fluctuating temperature at the larval stage they exhibit aberrations in retinal development [22]. The role of some miRNAs may be to add biological robustness during development or homeostasis by modulating gene regulatory networks [23]. For these reasons it is particularly important to understand the roles of miRNAs in stress response. 

Stress can originate biologically from within the organism (such as that caused by disease or abnormal cellular behaviour) or externally from non-biological sources (such as toxic agents or changes in the environment). In this study we have analysed the changes in miRNA levels that occur in response to the latter. Over the past decade a number of studies have been performed to profile changes in miRNA expression following insult with various environmental challenges. The results of such studies are often conflicting, and may be due to differences in the experimental setup. In order to make sense of this increasing pool of data a central resource is required which can be used to meta-analyse the results of these studies, confirm the identity of key miRNAs and infer novel biological roles for non-coding RNAs in stress. A database exists for miRNA responses following stress induction in plants [24]. However, a comprehensive database of such data with the functionality to perform useful meta-analyses has not been reported for other eukaryotes. Here we address this issue with a novel database and web tool which we call miRStress. This manually curated database contains more than 7,500 entries from over 300 publications. To validate the usefulness for biological discovery of this resource we used the database to meta-analyse the effects of various stress types, including hypoxia and radiation. The results confirm the identification of several miRNAs already known from functional studies to be directly involved in response to these stimuli. In addition, several other miRNAs are identified that have not previously been associated with these stresses. These results suggest the miRStress database is a useful new tool for understanding the biology of miRNAs. 

## Results and Discussion

### The miRStress database

There is a wealth of data in the literature showing the effect of environmental stresses on miRNAs, but it is scattered in a large number of disparate publications. Meta-analyses of this data would produce added insight into the molecular mechanisms of stress-response. To facilitate this process we manually curated the miRStress database, which describes the changes in miRNA levels following a varied array of stress types in eukaryotic cells. As of June 2013 the database contained more than 7,500 entries, annotated from 315 publications spanning seven years. An initial analysis of all the entries in the database reveals the miRNAs that are most frequently deregulated in response to all stress types (table 1). The miRNAs that are affected most often are miR-21, miR-210 and miR-34a. This is consistent with previous reports of clear roles for these miRNAs in DNA damage-response and hypoxia [16]. 

**Table 1:** miRStress-generated list of the most frequently deregulated miRNAs across all stress types.

**miRNA**	**Up**	**Down**	**NR**	**Sum**	**% up**	**% down**
**21**	68	25	1	94	72.3	26.6
**210**	72	10	1	83	86.7	12.0
**34a**	49	14	0	63	77.8	22.2
**17**	24	37	1	62	38.7	59.7
**16**	35	23	1	59	59.3	39.0
**125b**	30	25	1	56	53.6	44.6
**26a**	25	27	1	53	47.2	50.9
**20a**	19	27	1	47	40.4	57.4
**155**	33	14	0	47	70.2	29.8
**29a**	25	20	1	46	54.3	43.5

Columns indicate the miRNA name, the number of incidences where the miRNA is stimulated (up) or repressed (down) by the stress. NR indicated that the direction of change was not reported in the publication.

### Identification of miRNAs involved in radiation response

To demonstrate the potential of miRStress in identifying miRNAs with biological importance in stress response we analysed entries related to radiation. Several studies have attempted to measure the effects of radiation on miRNA levels. The degree of overlap between these studies is variable, due to the differences in radiation type, dose, cell type, miRNA measurement technique and other differences in experimental approach. Observing miRNAs that consistently change in response to radiation across many studies could imply they have greater functional importance. Some attempts have been made to collate and analyse these disparate publications [25,26], but a recent comprehensive meta-analysis of these studies has not been described. 

To identify radiation-related miRNAs we selected the radiation treatment group on the miRStress database. The database returned a list of miRNAs along with the number of reports of a miRNA being significantly deregulated. Table 2 shows the list of miRNAs whose level changes in at least ten instances in the database. The most frequently deregulated miRNAs were miR-21 and miR-34a. This is consistent with previous work showing a role for these miRNAs in the response to genotoxic stress, including radiation [1,27,28]. Indeed, many of the miRNAs in table 2, which we term ‘radiation-miRNAs’ have been previously shown to play a role in either the response to radiation or in conferring differential sensitivity to radiation. There are other miRNAs in table 2, including miR-15b and miR-19b, which have not been overtly identified as being related to radiation, suggesting that these miRNAs represent novel candidates for further study by the radiobiology field. With some of these miRNAs there are clues to their potential involvement in radiation response from other studies. For instance, the let-7 family are known to regulate a number of oncogenes, so specific members of the family may tip the balance between cell cycle arrest and apoptosis following irradiation [29]. Evidence suggests that miR-15b can regulate cell cycle progression [30] and apoptosis [31]. Interestingly, miR-19a/b are able to increase resistance of gastric cancer cells to chemotherapy by affecting drug efflux pathways and inhibiting apoptosis [32]. The finding that exosomes associated with RNA mediate the radiation-induced bystander-effect also hints at a role for miRNAs in the intercellular response to ionizing radiation [33]. Whilst there is much left to elucidate in the miRNA-mediated responses to radiation, the results from this study provide some strong candidates worthy of further characterisation. 

**Table 2:** miRStress-generated list of the most frequently deregulated miRNAs following radiation treatment.

**miRNA**	**Up**	**Down**	**NR**	**Sum**	**% up**	**% down**
**21**	11	6	1	18	61.1	33.3
**34a**	11	6	0	17	64.7	35.3
**16**	10	5	1	16	62.5	31.3
**17**	8	6	1	15	53.3	40.0
**let-7b**	5	9	1	15	33.3	60.0
**let-7g**	9	5	0	14	64.3	35.7
**let-7a**	5	8	1	14	35.7	57.1
**let-7f**	6	7	0	13	46.2	53.8
**19b**	6	5	1	12	50.0	41.7
**let-7d**	4	6	2	12	33.3	50.0
**let-7c**	7	5	0	12	58.3	41.7
**125b**	5	6	1	12	41.7	50.0
**143**	4	5	2	11	36.4	45.5
**24**	8	3	0	11	72.7	27.3
**20a**	4	6	1	11	36.4	54.5
**15b**	4	5	2	11	36.4	45.5
**106a**	3	6	1	10	30.0	60.0
**106b**	4	5	1	10	40.0	50.0
**let-7e**	4	6	0	10	40.0	60.0
**221**	8	2	0	10	80.0	20.0

Columns indicate the miRNA name, the number of incidences where the miRNA is stimulated (up) or repressed (down) by the stress. NR indicated that the direction of change was not reported in the publication.

In order to gain further insight into the potential roles of miRNAs returned by miRStress we used bioinformatics tools to analyse the functions of their predicted targets. The online miRNA binding-site prediction tool miRWalk [34] was used to produce a list of predicted gene targets for each of the radiation-miRNAs in table 2. miRWalk reports the result of various miRNA target-prediction algorithms, thereby allowing the user to estimate whether predicted targets are low- or high-confidence interactions. For each radiation-miRNA we obtained a list of high-confidence predicted gene targets (at least six different algorithms within miRWalk predict an interaction). To analyse the potential role of these predicted genes we used the DAVID functional annotation [35,36]. For each radiation-miRNA we generated a list of high-confidence ‘predicted’ KEGG pathways that are enriched in the target mRNAs. The predicted-pathways most commonly targeted are shown in in text S1. Interestingly, the most commonly predicted pathway was MAPK signalling, which has been previously observed as playing a role in radiation response [37]. To assess whether the predicted-pathways targeted by radiation-miRNAs underpin a genuine biological phenomenon, rather than a non-specific quirk obtained when any set of miRNAs is analysed, we repeated the analysis with an equivalent number of control miRNAs. The control miRNAs were chosen on the basis that they appear only once on the list of radiation-affected miRNAs and are therefore unlikely to represent genuine radiation-miRNAs. The pattern of predicted-pathways for the control-miRNAs is different to those obtained with radiation-miRNAs (text S1). Indeed, many of the predicted pathways (such as the MAPK signalling pathway) appear much more often for the radiation miRNAs than the control miRNAs, suggesting that they do represent a biological effect. 

**Figure 1:**
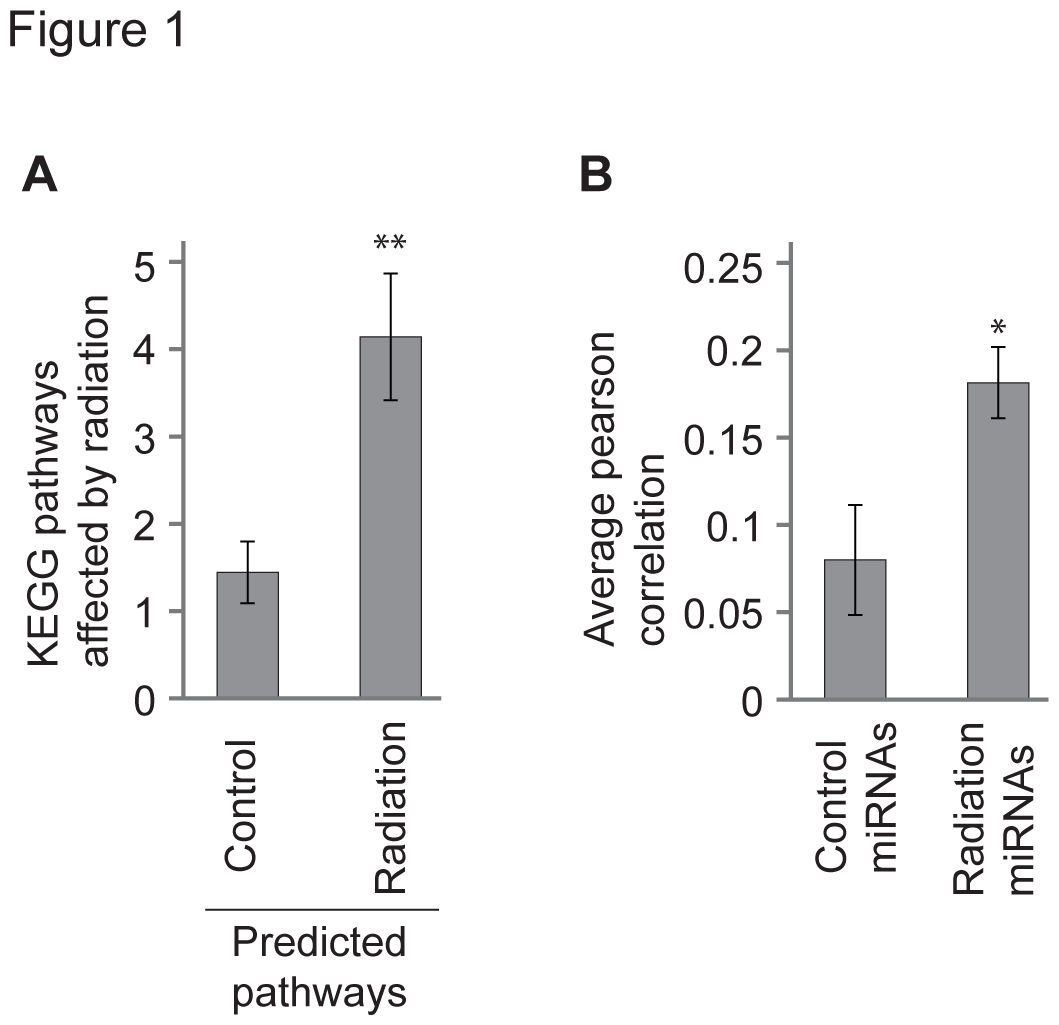
Radiation-responsive miRNAs predicted by miRStress are biologically relevant. A: The miRNAs which are most frequently deregulated following radiation stress, as reported by miRStress, were used to ‘predict’ radiation-responsive pathways, as described in the methods. Control pathways were selected by using a list of control miRNAs. These were then compared to a list of KEGG pathways which are observed (as opposed to predicted) to actually change following radiation (in a total of 18 datasets). The average frequency with which the predicted control or radiation pathways appears in the observed pathways is shown. On average the radiation predicted-pathways are more frequently present in the observed pathways (t test, p 0.002). In other words, the radiation miRNAs are predicted to target pathways which actually change following radiation. Error bars show standard error of the mean for 20 (for radiation) and 21 (for control) pathways. B: Radiation and control miRNAs used in part A were used to test for correlations between SF5 (surviving fraction of cells following a 5 Gray radiation dose) and miRNA level across the NCI-60 panel. A Pearson correlation was obtained for each radiation and control miRNA (not all were available on the microarray platform). The average Pearson correlation value (see methods) for the control and radiation miRNAs is shown. The radiation miRNAs have a significantly higher correlation with radiosensitivity compared to the control miRNAs (t test, p = 0.02).

In addition to the studies measuring the levels of miRNAs there have also been a number of publications describing changes in mRNA levels in response to radiation. We downloaded 18 sets of microarray data from such studies and identified genes deregulated by at least two-fold from each dataset. These genes were then analysed using DAVID and the pathways enriched in the datasets were counted and compared to the predicted pathways (text S1). Interestingly, MAPK signalling was enriched in half of the 18 datasets, consistent with the identification of MAPK signalling in the radiation miRNA predicted-pathways. Radiation predicted-pathways appeared on average 4.2 times in the actual radiation pathways. This was significantly more (t test, p = 0.002) than the control predicted-pathways, which appeared on average 1.5 times in the list of pathways that are actually deregulated in radiation response (Figure 1A). In other words, the miRNAs which are most frequently affected by radiation are ‘predicted’ to target the pathways which are ‘actually’ affected following irradiation. This is consistent with a role for the radiation-miRNAs in influencing gene expression following irradiation of cells. 

To further validate the biological relevance of the radiation miRNAs identified by miRStress we utilised previously published datasets featuring radiosensitivity and miRNA expression data across the NCI-60 panel. The NCI-60 panel is a collection of human cell lines derived from various cancer types. This panel has been well characterised at the molecular level [38], with data available on expression of miRNAs and mRNAs, as well as sensitivity to radiation and thousands of compounds. Specifically we used the SF5 (surviving fraction of cells after a 5 Gray dose of radiation) value for each cell line [39] and the levels of miRNA expression in the E-MTAB-327 dataset [40]. To test for relationships between miRNAs and radiation resistance we performed Pearson correlation analyses between miRNA levels and radiosensitivity across the panel. For each miRNA this yielded a Pearson correlation which is indicative of the strength of association between the level of that miRNA and the level of radioresistance. We then assessed whether the magnitude of the Pearson correlations for radiation miRNAs was, on average, significantly higher than those for the control miRNAs. Our results show that the magnitude of the average Pearson correlation for radiation miRNAs is indeed significantly higher (t test, p = 0.017) for the radiation miRNAs compared to control miRNAs (Figure 1B). A similar analysis using the coefficient of determination (R^2^) for these correlations showed that the average R^2^ value for radiation miRNAs was more than three times higher than that for control miRNAs (t test, p = 0.03). This suggests that the miRNAs identified by miRStress as being associated with radiation response are more likely to correlate with radiosensitivity than the control miRNAs. 

In order to be more confident in the accuracy of miRStress in calling genuine radiation-related miRNAs we performed a receiver operating characteristic (ROC) curve analysis. This method can be used to determine how good a tool is at taking a variable input (in this case the number of times a miRNA is de-regulated by a specific stress, as determined by miRStress) and a binary output (whether the miRNA really is involved in radiation). To determine whether a miRNA represents a true functional miRNA we analysed the literature. A true positive was categorised as a miRNA that had previously been shown to be functionally involved in radiation response, either by being involved in a defined radiation response pathway or by affecting radiation resistance when manipulated. To perform the ROC curve analysis we used the list of radiation miRNAs (in table 2) and the same control miRNAs used in the previous analyses (that appear only once in the miRStress list of radiation-de-regulated miRNAs). The analysis resulted in an area under the curve (AUC) of 0.857, which is indicative of a good test. One note of caution is that in this analysis the assignment of ‘true positives’ is based on appearance in the literature (which for the purposes of this test we take at face value). However, just because a miRNA does not appear in the literature as a functional radiation miRNA does not mean that this miRNA definitely does not play a role, which makes it more difficult to accurately determine the binary input for the ROC test. Nevertheless, the high AUC of 0.857 is consistent with a good sensitivity and specificity of the miRStress tool in determining miRNAs involved in stress. Taken together these results confirm that the data produced by miRStress are of biological relevance, and that the program can identify new candidate miRNAs that may be involved in response to specific stressors. 

### Novel miRNAs in hypoxia

Hypoxia, which occurs in cells exposed to lower levels of oxygen, is extensively studied as it is a key feature of tumour progression and chemotherapy response [41].. Hypoxia is also associated with the pathology of ischemic disorders, including myocardial infarction and stroke [42]. As with many other biological processes, miRNAs have been shown to play a role in mediating the response to hypoxia [23]. Having validated the miRStress database we next wished to identify novel roles for miRNAs in response to hypoxia. The most frequently deregulated miRNAs in hypoxia response, which we term ‘hypoxia miRNAs’, are shown in table 3. In contrast to radiation miRNAs, which are more variably up- or down-regulated by radiation, the hypoxia miRNAs appear much more likely to be induced by hypoxic stress. 

**Table 3:** miRStress-generated list of the most frequently deregulated miRNAs following hypoxia treatment.

**miRNA**	**Up**	**Down**	**NR**	**Sum**	**% up**	**% down**
**210**	56	0	0	56	100	0
**21**	12	2	0	14	85.7	14.3
**155**	8	3	0	11	72.7	27.3
**181b**	8	3	0	11	72.7	27.3
**26b**	9	1	0	10	90.0	10.0
**106a**	9	0	0	9	100	0
**26a**	8	0	0	8	100	0
**213**	8	0	0	8	100	0
**192**	8	0	0	8	100	0

Columns indicate the miRNA name, the number of incidences where the miRNA is stimulated (up) or repressed (down) by the stress. NR indicated that the direction of change was not reported in the publication.

A key regulator of the hypoxic response is miR-210, which is capable of regulating various pathways including cell cycle, apoptosis and oxidative metabolism [43]. The prominence of this miRNA was confirmed by our miRStress analysis which identified miR-210 as by far the most frequently deregulated miRNA in response to hypoxia. Hypoxia-inducible factors (HIF) are transcription factors that regulate various genes involved in response to hypoxia, including miR-210 [41,43]. Regulating the levels of HIFs, and in particular HIF-1α, is critical to an appropriate response during low oxygen tension. Various miRNAs have been shown to negatively regulate HIF-1α, including miR-155 [44], another miRNA that was identified by the miRStress analysis of hypoxia-responsive miRNAs. As described above, miR-21 has been implicated in numerous stress responses and is able to confer resistance to hypoxia by regulating the tumour suppressor PTEN [45]. Several miRNAs, including miR-20a/b and miR-424, which have been reported to affect hypoxia response [23,43,46], did not appear in the top ten hypoxia miRNAs identified in the miRStress analysis. Instead a number of other miRNAs (miR-181b/c, miR-213, miR-26a/b, miR-106a and miR-192), which have no obvious connection to hypoxia in the literature, are more frequently deregulated. This does not rule out a role for previously identified hypoxia-related miRNAs, but suggests that other miRNAs may have an equally important role in hypoxia response. Interestingly, according to the miRStress analysis, miR-181b is deregulated almost as frequently as miR-21 in hypoxia. NF-κB has been shown to be involved hypoxia response [47], and miR-181b is able to regulate vascular inflammation by directly targeting NF-κB [48]. Glycerol-3-phosphate dehydrogenase 1-like (GPD1L) is repressed by miR-210 under hypoxic conditions, which leads to stabilisation of HIF-1α [49]. Analysis using the miRNA-mRNA target prediction algorithm miRWalk suggests that miR-181b may also target GPD1L and thus stabilise HIF-1α. miR-26a/b have been shown to be involved in cancer progression by targeting the cell cycle or apoptosis [49,50]; whether miR-26a/b act on oncogenes or tumour suppressors is unclear, and the conflicting reports in the literature suggest that the effects of miR-26a/b are context-dependent. Indeed, the finding that miR-26a plays a role in oxidative stress response via apoptotic signalling suggests a potential role in hypoxic response [51].The ROC curve analysis performed on the hypoxia miRNA list shows an AUC of 0.859, suggesting that the tool is consistent in producing accurate lists of biologically relevant miRNAs in hypoxia. It also suggests that the accuracy of the tool is consistent for different stresses. Whilst further experimental evidence is needed to confirm the role (if any) and pathways of these miRNAs, our results nevertheless have uncovered several novel candidates for potential involvement in the hypoxic response. 

The ability of miRStress to identify useful candidates will in part depend on how many reports have been published using a particular stress. At the time of submission the miRStress database included 491 incidences of stress treatment. Most of the >170 specific treatments were performed once, twice or three times. However, 23 specific treatments were reported in at least five different instances, of which there are ten stress types that were performed on nine different occasions. Therefore there are a number of stress types that are amenable to a useful meta-analysis using miRStress. For stress-types with fewer publications the accuracy of the tool in predicting genuinely functional miRNAs and stress biomarkers will most likely be lower. However, given that we have shown that miRStress works accurately for well-studied stress types, such as radiation and hypoxia, it follows that as the database is updated and the number of included publications grows, so will the ability of this tool to identify biologically relevant miRNAs and stress biomarkers for more stress types. 

There are a number of potential biases which must be considered when analysing the miRStress output. There will be an element of publication bias as only English-language articles were included, and research with seemingly negative results may have been withheld from publication. There is also a degree of bias within the database caused by the methodology used in the different publications. Some use more ‘open’ platforms such as RNA-seq, microarrays or high-throughput PCR-panels, whereas others only include a small number of primers to test specific miRNAs by PCR. Even within the more open platforms there is bias; for example, different microarray platforms contain different selections of miRNAs. Early discoveries in the field can also lead to subsequent bias. A good example of this is miR-210, which was shown in 2008 to be an important player in hypoxia [52]. Since then a number of articles have used PCR to confirm the induction of miR-210 without testing the effect on many other miRNAs. This is reflected in the miRStress analysis which shows a very high number of instances of miR-210 deregulation following hypoxia. This high number relative to other miRNAs is in part due to the bias described above. The database is therefore more likely to produce false negatives than false positives. Nevertheless, as we have shown for miRNAs involved in hypoxia and radiation, it is possible to identify miRNAs with previously undiscovered roles in stress response. 

The miRStress analysis has therefore uncovered the importance of hitherto under-appreciated miRNAs in the processes of hypoxia and radiation response, and will be a useful tool for researchers studying the effects of stress response. Future work should unravel the precise roles and mechanisms of the miRNA candidates uncovered by miRStress. 

## Materials and Methods

### Study selection

The search term ‘microRNA’ was entered into PubMed to obtain a list of all microRNA publications to date. The entire history of microRNA publication abstracts (>20,000 publications) were manually searched for any abstracts mentioning differential regulation of microRNAs following any stress treatment of cells. If the abstract did not specifically mention the use of a stressor followed by miRNA measurement then it was not included. Should users encounter a paper which should be included in the database but has been omitted then they are encouraged to contact the corresponding author (DRFC). We did not include reports of treatments related to biological stresses, such as disease, infection with viruses or bacteria, or treatment with biological macromolecules such as hormones and peptides. For ease of interpretation we also excluded combination treatments. For inclusion in the database the miRNA changes needed to be indicated as statistically significant. At the time of manuscript preparation a total of 7,663 miRNA entries from 315 papers were included in the database. For each paper we manually annotated various details, including the cell type, stressor conditions, quantification methods and miRNAs that were deregulated. 

### Database construction

The miRStress database is stored in a plain ascii flat file format, and a module to interrogate it was constructed using the Python programming language (http://www.python.org). Additional Python modules were written utilising “pyro” (https://pypi.python.org/pypi/Pyro4) and the moinmoin wiki framework (http://moinmo.in) to build the web interface. Interested readers should contact DRFC or MGP concerning the availability of the software. The database is hosted on the Cell Systems Modelling Group website and is freely accessible at http://mudshark.brookes.ac.uk/MirStress. 

Data is accessed by browsing the different stress types. Clicking on a given treatment group or a specific treatment loads the miRNA information into the results output page. These results can then be accessed as a list of miRNA frequencies (by clicking on the browse RNAs option) or a list of publications (by clicking on browse PMIDs [Pubmed IDs]). The number of ‘reports’ describes the number of publications in which the selected stress appears. The output also includes the number of incidences within those reports in which the given miRNA is increased or decreased. If a paper describes multiple readings for a given miRNA in a cell line (for example at different time points, or different stress doses) then these not considered to be multiple incidences (so would only add one to the incidence count). 

In addition to the web interface a more flexible downloadable version of miRStress is available at https://sourceforge.net/projects/mirstress/. The miRStress download module is also written in python and allows users to search the database whilst offline. The download module is powered using the same python script as the online miRStress website with a separate tkinter script used to form the graphical user interface. 

### Radiation miRNA validation

For each of the radiation and control miRNAs a list of high confidence targets were identified using the online miRNA binding-site prediction tool miRWalk [25]. This tool performs a form of meta-analysis, comparing the results of various other miRNA-target prediction algorithms. We selected genes that were predicted to be targets by at least 6 of these algorithms. The DAVID functional annotation tool was then used to identify KEGG pathways that are enriched in the list of predicted gene targets [35,36]. We labelled these as ‘predicted pathways’. If a given KEGG pathway was ‘predicted’ to be targeted by at least three radiation miRNAs and at least 50% fewer control miRNAs then we considered this to be a ‘radiation pathway’. Otherwise it was labelled as a ‘control pathway’. These criteria were selected arbitrarily to reflect the heterogeneity in radiation response pathways (as well as heterogeneity of miRNA targeting) and the requirement for radiation pathways to be more abundant than random pathways. 

Eighteen expression microarray datasets documenting mRNA changes following ionizing radiation treatment were obtained from Gene Expression Omnibus. Datasets were individually imported into Genespring 12.5 and normalised using Robust Multi-array Average. Each dataset was then normalised to the median value for that dataset. Genes whose expression was altered by at least 2-fold in irradiated compared to control samples were imported into DAVID Bioinformatics Resource 6.7 [35,36]. This allowed identification of KEGG pathways that were significantly enriched in each of the 18 radiation datasets. 

SF5 (surviving fraction of cells following a 5 Gray dose of gamma rays) data were obtained for each cell line in the NCI-60 panel from previously published results [39]. Levels of miRNA expression for each cell line were obtained from the E-MTAB-327 dataset [40]. Pearson correlations were obtained between each miRNA and the SF5 data across the panel of cell lines. For comparison of different miRNAs the magnitude of Pearson correlation values was obtained by converting any negative values into positives. 

### Receiver operating characteristic (ROC) analysis

The ROC analysis was performed using SPSS (v19). In each test the list of high-confidence miRNAs for a given stress (either radiation or hypoxia, see tables 2 and 3, respectively) was compared to an equivalent number of control miRNAs (which only appear once in miRStress for that given stress). For the test variable we used the number of appearances in the miRStress database. For the state variable we used a dichotomous output of whether the miRNA was a ‘true positive’ or not. We defined a miRNA as a true positive if that miRNA had previously been shown to be functionally involved in the stress response, either by being involved in a defined stress response pathway or by affecting resistance (to the given stress) when manipulated. 

## Supporting Information

Text S1 **Analysis of the actual and predicted (based on miRNA deregulation) pathways following radiation treatment.**
(DOCX)
