# Supplementary material for: Meta-Analysis Using a Novel Database, miRStress, Reveals miRNAs That Are Frequently Associated with the Radiation and Hypoxia Stress-Responses
Source: PLoS One. 2013 Nov 14;8(11):e80844. doi: 10.1371/journal.pone.0080844 (PMC3828287; doi:10.1371/journal.pone.0080844)
Supplement: Text S1 — Analysis of the actual and predicted (based on miRNA deregulation) pathways following radiation treatment. (DOCX) [file pone.0080844.s001.docx]

Analysis of the actual and predicted (based on miRNA deregulation) pathways following radiation treatment. Columns indicate the KEGG pathway name, the number of radiation miRNAs predicted to target this pathway, the number of control miRNAs predicted to target this pathway, whether or not the pathway is designated as a ‘control pathway’ or ‘radiation pathway’ (see materials and methods), and the number of times these pathways are deregulated in 18 mRNA datasets (in which cells were irradiated).

| Name of KEGG pathway | Number of radiation miRNAs that are predicted to target the pathway | Number of control miRNAs that are predicted to target the pathway | Predicted Pathway label | Number of studies in which this pathway was actually deregulated |
| --- | --- | --- | --- | --- |
| MAPK signaling pathway | 16 | 4 | Radiation pathway | 9 |
| Focal adhesion | 12 | 6 | Radiation pathway | 5 |
| Endocytosis | 11 | 4 | Radiation pathway | 2 |
| Axon guidance | 9 | 5 | Radiation pathway | 3 |
| TGF-beta signaling pathway | 8 | 3 | Radiation pathway | 5 |
| ECM-receptor interaction | 7 | 0 | Radiation pathway | 5 |
| Lysosome | 6 | 0 | Radiation pathway | 1 |
| Melanogenesis | 6 | 4 | Radiation pathway | 2 |
| ErbB signaling pathway | 5 | 3 | Radiation pathway | 5 |
| Fc epsilon RI signaling pathway | 4 | 1 | Radiation pathway | 0 |
| Progesterone-mediated oocyte maturation | 4 | 2 | Radiation pathway | 1 |
| T cell receptor signaling pathway | 4 | 1 | Radiation pathway | 3 |
| Calcium signaling pathway | 4 | 0 | Radiation pathway | 5 |
| Neurotrophin signaling pathway | 3 | 1 | Radiation pathway | 1 |
| Aldosterone-regulated sodium reabsorption | 3 | 1 | Radiation pathway | 1 |
| VEGF signaling pathway | 3 | 1 | Radiation pathway | 2 |
| Apoptosis | 3 | 0 | Radiation pathway | 5 |
| Oocyte meiosis | 3 | 2 | Radiation pathway | 6 |
| Cell cycle | 3 | 1 | Radiation pathway | 10 |
| p53 signaling pathway | 3 | 1 | Radiation pathway | 12 |
| Adherens junction | 5 | 4 | Control pathway | 2 |
| Wnt signaling pathway | 5 | 6 | Control pathway | 2 |
| Gap junction | 4 | 3 | Control pathway | 3 |
| mTOR signaling pathway | 3 | 3 | Control pathway | 3 |
| GnRH signaling pathway | 2 | 2 | Control pathway | 0 |
| D-Glutamine and D-glutamate metabolism | 2 | 0 | Control pathway | 0 |
| Dorso-ventral axis formation | 2 | 1 | Control pathway | 0 |
| Limonene and pinene degradation | 2 | 0 | Control pathway | 0 |
| Fc gamma R-mediated phagocytosis | 2 | 1 | Control pathway | 0 |
| Toll-like receptor signaling pathway | 2 | 0 | Control pathway | 1 |
| Nitrogen metabolism | 2 | 1 | Control pathway | 1 |
| Vascular smooth muscle contraction | 2 | 2 | Control pathway | 2 |
| B cell receptor signaling pathway | 2 | 0 | Control pathway | 2 |
| Insulin signaling pathway | 2 | 5 | Control pathway | 3 |
| Purine metabolism | 1 | 0 | Control pathway | 0 |
| Ubiquitin mediated proteolysis | 1 | 1 | Control pathway | 0 |
| SNARE interactions in vesicular transport | 1 | 0 | Control pathway | 0 |
| Alanine, aspartate and glutamate metabolism | 1 | 0 | Control pathway | 0 |
| Arginine and proline metabolism | 1 | 0 | Control pathway | 0 |
| Lysine degradation | 1 | 0 | Control pathway | 0 |
| Notch signaling pathway | 1 | 2 | Control pathway | 0 |
| Glycosphingolipid biosynthesis | 1 | 1 | Control pathway | 0 |
| Selenoamino acid metabolism | 1 | 0 | Control pathway | 0 |
| beta-Alanine metabolism | 1 | 0 | Control pathway | 0 |
| Heparan sulfate biosynthesis | 1 | 1 | Control pathway | 1 |
| Long-term potentiation | 1 | 3 | Control pathway | 3 |
| Phosphatidylinositol signaling system | 1 | 1 | Control pathway | 3 |
| Regulation of actin cytoskeleton | 1 | 2 | Control pathway | 3 |
| Adipocytokine signaling pathway | 1 | 0 | Control pathway | 4 |
| Chemokine signaling pathway | 1 | 1 | Control pathway | 4 |
| Cytokine-cytokine receptor interaction | 1 | 1 | Control pathway | 8 |
